# Extreme conservation of noncoding DNA near *HoxD *complex of vertebrates

**DOI:** 10.1186/1471-2164-5-75

**Published:** 2004-10-06

**Authors:** Chilaka Sabarinadh, Subbaya Subramanian, Anshuman Tripathi, Rakesh K Mishra

**Affiliations:** 1 Centre for Cellular and Molecular Biology, Uppal Road, Hyderabad 500 007, India

## Abstract

**Background:**

Homeotic gene complexes determine the anterior-posterior body axis in animals. The expression pattern and function of *hox *genes along this axis is colinear with the order in which they are organized in the complex. This 'chromosomal organization and functional correspondence' is conserved in all bilaterians investigated. Genomic sequences covering the *HoxD *complex from several vertebrate species are now available. This offers a comparative genomics approach to identify conserved regions linked to this complex. Although the molecular basis of 'colinearity' of *Hox *complexes is not yet understood, it is possible that there are control elements within or in the proximity of these complexes that establish and maintain the expression patterns of *hox *genes in a coordinated fashion.

**Results:**

We have compared DNA sequence flanking the *HoxD *complex of several primate, rodent and fish species. This analysis revealed an unprecedented conservation of non-coding DNA sequences adjacent to the *HoxD *complex from fish to human. Stretches of hundreds of base pairs in a 7 kb region, upstream of *HoxD *complex, show 100% conservation across the vertebrate species. Using PCR primers from the human sequence, these conserved regions could be amplified from other vertebrate species, including other mammals, birds, reptiles, amphibians and fish. Our analysis of these sequences also indicates that starting from the conserved core regions, more sequences have been added on and maintained during evolution from fish to human.

**Conclusion:**

Such a high degree of conservation in the core regions of this 7 kb DNA, where no variation occurred during ~500 million years of evolution, suggests critical function for these sequences. We suggest that such sequences are likely to provide molecular handle to gain insight into the evolution and mechanism of regulation of associated gene complexes.

## Background

Eukaryotic genome contains a large excess of non-coding sequences. Conservation of these sequences among species is a strong indication of their functional significance. With the availability of genome sequences it is possible to identify such sequences taking a comparative genomics approach [1-4]. The clusters of homeotic genes, which are expressed in a coordinated manner [5], are among the most conserved regions of the vertebrate genome. Clustering of genes that are regulated in a linked manner has been noticed in several other cases [6,7]. However, the molecular mechanism behind such coordination in regulation is not yet understood. Several mechanisms have been proposed that link the organization of homeotic genes and the spatio-temporally controlled expression [8]. Colinearity in *hox *complexes was first discovered in *Drosophila *[9] and later studies on the bithorax complex have demonstrated the role of chromatin organization in its regulation [10]. Recent studies on the *HoxD *complex suggest a role for higher order chromatin organization in the regulation of this complex involving up to 20 kb upstream region [11].

## Results and discussion

**Figure 1:**
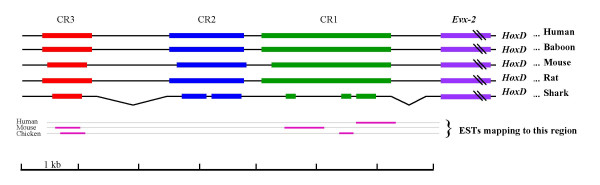
**Schematic representation of sequence conservation in the *HoxD *upstream region. **Human sequence (AC009336; from position 56601 to 64095) was compared to the corresponding sequences of *Papio hamadryas *(AC116665), *Heterodontus francisci *(AF224263), *Mus musculus *(AC015584), *Fugu rubripes *(CAAB01000449) and *Rattus norvegicus *(NW_042732). Sequences that are conserved across vertebrates are shown as blocks. The conservation extends beyond these blocks within primates and rodents. ESTs found in the database corresponding to this region are also shown. ESTs mapping to CR3 are BB838602 from mouse 8 cell embryo and BU129154 from chicken 36 stage limb; and those mapping to CR1 are AA620964 from human testis; BB332383, BB335110, BB334358, BB333569 from 6 and10 days mouse neonate medulla oblongata and BU255316 from chicken 36 stage limb.

**Figure 2:**
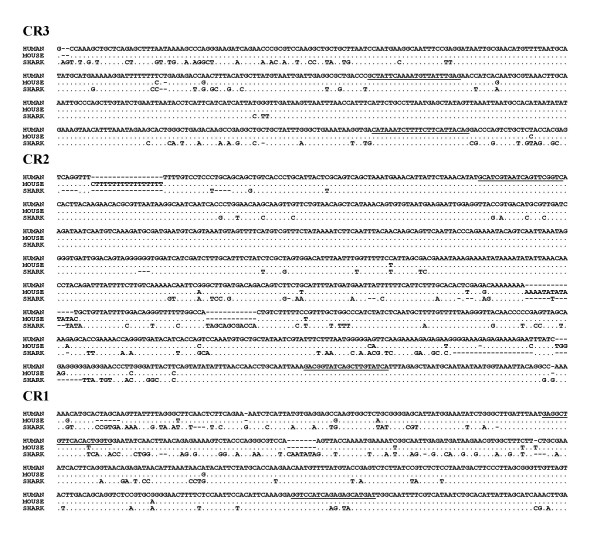
**Comparison of conserved regions from human, mouse and shark. **Conserved bases of mouse and shark are shown as '.' and '-' indicates indels. Underlined sequences of human indicate primers that were used for amplification of the corresponding sequence from different vertebrates.

We compared genomic regions flanking *hox *complexes in order to identify conserved regions with potential regulatory function. Here we report that the upstream regions of *HoxD *complexes of human, mouse, rat, sacred baboon, horn shark, zebra fish and puffer fish contain long stretches of extremely conserved sequences. In the 25 kb region upstream of the *HoxD *complex from these organisms we found an extremely conserved region spread in three blocks located within 7 kb from the 3' end of the *Evx-2 *gene. These conserved regions, designated as Conserved Region 1, Conserved Region 2 and Conserved Region 3 (CR1, CR2 and CR3) (Fig. 1) show a degree of conservation not seen before among distant species. Detailed analysis of each region spanning to several hundred base pairs, in particular the CR2 shows several stretches of 100 % conservation, Fig. 2. We also noticed longer stretches of conservation among mammals, which gradually shortens as we go towards lower vertebrates, defining the core of each conserved region, across the vertebrate classes, see Additional file 1. This and the fact that in case of shark, as compared to mammals, the intervening sequence lengths between CR2 and CR3, and CR1 and *Evx-2 *is shorter by ~1300 bp and ~600 bp, respectively (Fig. 1) suggest that starting from the shorter conserved regions, additional unique sequences have progressively been acquired and conserved during the evolution of primates from lower vertebrates. This may reflect the molecular basis of conservation and elaboration of *Hox *gene regulation during evolution of these species [12].

**Figure 3:**
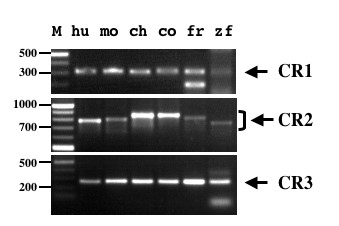
**Conservation of CR1, CR2 and CR3 in all vertebrate classes. **PCR amplification of different vertebrate genomic DNA samples using primers designed based on the human sequence. Lanes: M – size marker indicated in bp, hu – human, mo – mouse, ch – chicken, co – cobra, fr – frog and zf – zebra fish. The arrows indicate the corresponding products that have been confirmed by direct sequencing as well as Southern hybridization using human CRs as probe.

Universal occurrence of these sequences in all vertebrate classes was confirmed by their amplification using primers from human *HoxD *complex (Figure 3) followed by Southern hybridization and sequencing (unpublished observation). Furthermore, using CR1, CR2 or CR3 as query we searched genomic sequences of variety of eukaryotes in available databases. This search indicated that these sequences are single copy and vertebrate specific. While these conserved regions appear to be a key component of the *HoxD *complex of all vertebrates looked at, we did not find such a degree of conservation in the flanking regions of other *hox *complexes (*HoxA*, *B *and *C*) of vertebrates. In order to trace back the evolutionary origin of such sequences, it will be of interest to investigate occurrence of these sequences at the corresponding region in the hox complexes of species of urochordata, cephalochordata or even agnatha. In the tunicate *Oikopleura dioca*, where hox genes are dispersed but the spatial pattern seen in other animals is still present [13], we did not find CR1, CR2 or CR3. Also, we did not find any significant conserved region corresponding to these CRs in the amphioxus genomic region that contains the cluster of hox genes. It appears, therefore, that these extremely conserved sequences have originated in the vertebrates where the hox complex has additional distinct features of tight clustering compared to the insect hox clusters and the temporal colinearity, not seen in invertebrates.

Several recent reports using comparative genomics approach have identified conserved non-coding regions among different vertebrates [14-16] but none to the degree that we report here. The mechanism that may require such a high degree of conservation is not known. It is not, therefore, immediately clear what precisely is the role of these sequences. EST database search revealed that part of CR1 and CR3 are transcribed without any significant ORF but no EST corresponding to CR2 or any other part of the 7 Kb region was found, Fig. 1. A possible mechanism could involve RNA from this region that may function by base pairing to the genomic target sites. If that is the case, such high conservation could be expected. Role of transcription in the regulation of bithorax complex is emerging from recent studies [17].

## Conclusions

While such an extreme conservation of several hundred nucleotides over half a billion years in a region that does not code for any known proteins certainly implicates essential role for such sequences, probably in the regulation of *HoxD *complex, no known regulatory element requires such extreme conservation extending up to hundreds of base pairs. It is, therefore, likely that these elements could be components of a novel mechanism common to all vertebrates that regulates this gene complex. We are tempted to suggest that such a strongly conserved region from fish to human linked to a gene complex that is known to determine body axis formation may be the key determinant of molecular basis of early ontogeny. Early embryos of all vertebrates show striking similarity and we suggest that these elements may control the early expression pattern of *HoxD *which leads to similar pattern of the embryo shape. The gradient of conservation seen in this region from fish to human may further signify the evolutionary history of this locus and diversification of the morphological features along the anterior-posterior body axis of the vertebrate classes.

## Methods

### Sequence analysis

The genomic sequences that contained *Evx-2 *and any of the *Hoxd *genes were downloaded and annotated using gene/ORF prediction tools. Similar approach was used for other *hox *complexes. Homology searches of the upstream sequences of *HoxD *region from human (AC009336; from nucleotide 56601 to 64095) was carried out using the BLAST program of NCBI. The sequences that showed significant homology were further used to analyze the extent of homology by BLAST 2 program. The conserved regions from each sequence was obtained and subjected to multiple sequence analysis using Clustal X. In order to identify the expressed sequences corresponding to the conserved sequence, the conserved sequences along with the unique sequences were BLASTed against EST databases (human, mouse and dbEST).

The contigs that showed significant homology to the upstream sequences of human *HoxD *were annotated using the tBLASTx program and searching the translated amino acid sequence in the Swissprot database. Repeat masker program was used to look for repeat content. Genebank sequences used in this study are as follows: AC116665 *Papio hamadryas*, AF224263 *Heterodontus francisci*, AC015584 *Mus musculus*, AC009336 *Homo sapiens*, CAAB01000449 *Fugu rubripes *and NW_042732 *Rattus norvegicus*.

### DNA isolation, PCR amplification, sequencing and Southern hybridization

For the isolation of genomic DNA blood samples of human, chick and cobra (*Naja naja*) were used while liver tissue of mouse and muscle tissue of frog (*Bufo melanostictus*) and zebra fish were used. Standard protocol of DNA isolation was followed which included lysis, RNase A and proteinase K digestions followed by phenol/chloroform extraction and precipitation. Concentration and quality of the genomic DNA was checked on 0.7% agarose gel and UV absorption spectrophotometry. Based on the sequence of conserved regions primers were designed to amplify the three regions CR1, CR2 and CR3.

Primers used in this study to amplify conserved regions from different vertebrate species were:CR1 forward- GAGGCTGTTCACACTGGTGG,CR1 reverse- ATCATGCTCTCTGATGGACC,CR2 forward- GCATCGTAATCAGTTCGGTC,CR2 reverse- TGATACAAGCTGATACCGTC,CR3 forward- GCTATTCAAAATGTTATTTGAG and CR3 reverse- CTGTAATGAAGAAAAGATTTATG.

The 25 μl reaction was performed using 100 ng template DNA and 5 pmol each of forward and reverse primers. PCR protocol was as follows: initial denaturation step of 94°C for 3 min was followed by 35 cycles of 94°C for 1 min, 57°C for 1 min and 72°C for 1.30 min and final extension step at 72°C for 7 min. Authenticity of the PCR products was confirmed by direct sequencing and Southern hybridization, using the corresponding human DNA as probe.

### Note

An earlier version of this article was deposited in the 'Deposited Research' section of Genome Biology, , [18]. While this manuscript was in reviewing process, a report comparing human genome to several other mammalian sequences identified many highly conserved noncoding sequences [19]. Interestingly, this study also identified CR2 as uc.108 near *HOXD *and, in agreement to our observation, noted only a "core" conserved region in fish, suggesting that additional parts of the ultraconserved region were innovations after the common ancestor with fish.

## Authors' contributions

CS carried out the sequence analysis, PCR amplification and Southern analysis. SS participated in sequence analysis and DNA isolation from several organisms. AT carried out the sequencing of PCR products and participated in the sequence alignments. RKM conceived of the study, and participated in its design and coordination. All authors read and approved the final manuscript.

## Supplementary Material

Additional File 1 **Size and degree of conservation of CR1, CR2 and CR3 in different vertebrates. **Core of conserved regions and extended conserved regions between indicated species is shown as length of sequence and degree of conservation. Non-overlapping blocks of vertebrate conservation is indicated based on human, baboon, rat, mouse and shark comparison. Click here for file
