# Supplementary material for: Extreme conservation of noncoding DNA near HoxD complex of vertebrates
Source: BMC Genomics. 2004 Oct 6;5:75. doi: 10.1186/1471-2164-5-75 (PMC524357; doi:10.1186/1471-2164-5-75)
Supplement: Additional File 1 — Size and degree of conservation of CR1, CR2 and CR3 in different vertebrates. Core of conserved regions and extended conserved regions between indicated species is shown as length of sequence and degree of conservation. Non-overlapping blocks of vertebrate conservation is indicated based on human, baboon, rat, mouse and shark comparison. [file 1471-2164-5-75-S1.doc]

#### Size and degree of conservation of CR1, CR2 and CR3 in different vertebrates.

#### CR1 CR2 CR3

##### Core (bp) Extended (bp) Core (bp) Extended (bp) Core (bp) Extended (bp)

human/ baboon 317 [99%] 3119 [95%] 800 [99.4%] 4400 [95%] 256 [99.6%] included in CR-2

human/rat 317 [98.4%] 837 [91%] 800 [97.8] 1127 [95%] 256 [99.2%] 748 [94%]

human/mouse 317 [98.4%] 846 [92%] 800 [97.8%] 568 [98%] 256 [99.6%] 652 [97%]

556 [93%]

mouse/rat 317 [98.7%] 2180 [95%] 800 [98%] 1879 [92%] 256 [99.6%] 855 [81%]

459 [94%] 964 [94%] 483 [90%]

534 [90%]

human/shark 317 [90.2%] 253 [88%] 800 [93%] 581 [92%] 256 [93%] 414 [90%]

137 [85%] 175 [88%]

vertebrate 43 & 30 [100%] 144, 94, 59, 53 91, 62, 35 &

197 [93%] & 46 [100%] 62 [100%]

463 [96%]
